# Covered Stent of the Left Common Carotid and Subclavian Arteries Assist the Invasive Tumor Resection

**DOI:** 10.1155/2020/8882080

**Published:** 2020-12-07

**Authors:** Yo Tsukamoto, Takeo Nakada, Soichiro Fukushima, Mitsuo Yabe, Naoki Toya, Tadashi Akiba, Takashi Ohtsuka, Takao Ohki

**Affiliations:** ^1^Department of Surgery, Jikei University Kashiwa Hospital, 163-1 Kashiwashita, Kashiwa, Chiba 277-8567, Japan; ^2^Department of Surgery, The Jikei University School of Medicine, Nishishinbashi 3-19-18, Minatoku, Tokyo 105-8471, Japan

## Abstract

**Background:**

Some recent reports have described the usefulness of thoracic aortic stent grafts to facilitate en bloc resection of tumors invading the aortic wall. We report on malignant peripheral nerve sheath tumor resection in the left superior mediastinum of a 16-year-old man with neurofibromatosis type 1. The pathological margin was positive at the time of the first tumor resection, and radiation therapy was added to the same site. After that, a local recurrence occurred. The tumor was in wide contact with the left common carotid and subclavian arteries and was suspected of infiltration. After stent graft placement of these arteries to avoid fatal bleeding and cerebral ischemia by clamping these arteries and bypass procedure, we successfully resected the tumor without any complications.

**Conclusion:**

s. Here, we report the usefulness of the prior covered stent placement to aortic branch vessels for the resection of invasive tumor.

## 1. Background

Some recent reports have described the usefulness of thoracic aortic stent graft placement for the en bloc resection of malignant tumors that had invaded the thoracic aortic wall [1–4]. In this report, we could resect a superior mediastinal recurrent malignant tumor successfully, which was earlier irradiated with total of 60 Gy, using the covered stents in the left common carotid artery (LCCA) and the left subclavian artery (LSA). Covered stent placement in the aortic branch vessels was extremely useful in this case to avert catastrophic intraoperative bleeding. Herein, we describe an effectiveness of the prior stent graft placement to the aortic branch vessels as a new surgical indication. We propose a stent graft-assisted thoracic surgery, because it may help expand operative indications for patients who are otherwise deemed as unsuited to undergo surgical replacement of major arteries.

## 2. Case Presentation

**Figure 1:**
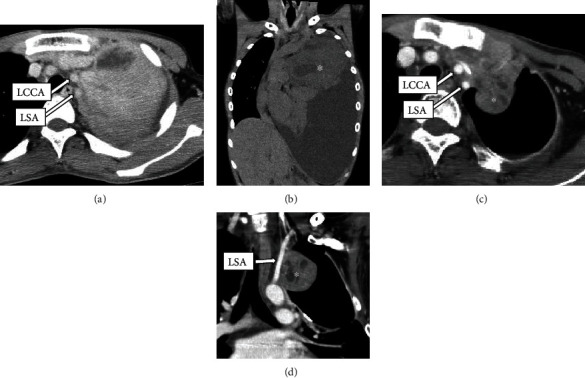
Before initial operation: (a) horizontal view, (b) frontal view. Chest computed tomography (CT) radiograph showing a left superior mediastinal tumor (^∗^) measuring 123 × 120 × 88 mm in size with massive pleural effusion. Before third operation: (c) horizontal view, (d) frontal view. Follow-up chest CT revealing local recurrence with rapid growth in the left superior mediastinum. The tumor had attached to the left common carotid artery (LCCA) and the left subclavian artery (LSA).

**Figure 2:**
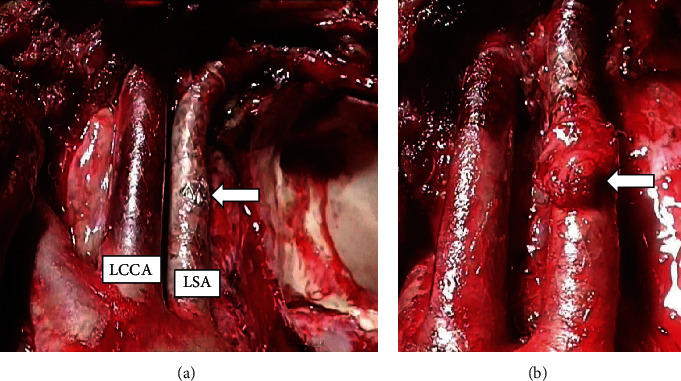
Intraoperative view. (a) Resected wall of the left subclavian artery (LSA), one-third of the circumference, causing no bleeding (arrow). (b) The resected portion was covered with a pericardial patch (arrow).

**Figure 3:**
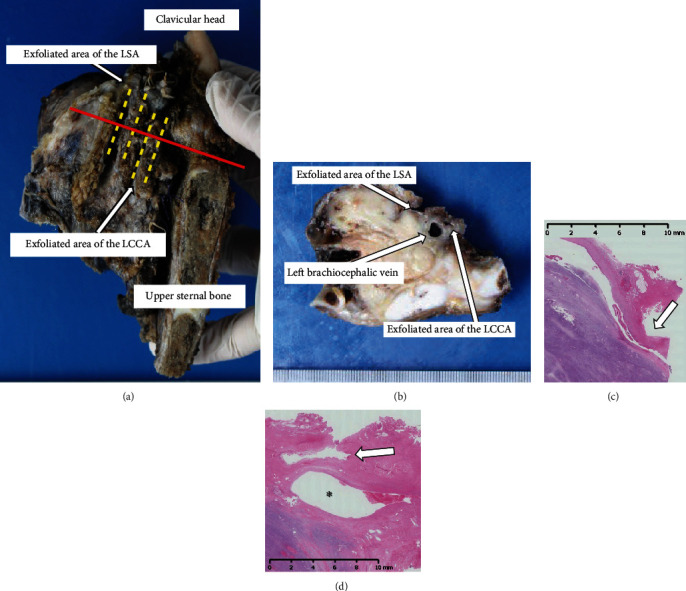
(a) The resected specimen (mediastinal view); (b) the cut surface on the red line of (a). (c) Microscopically, the fibrotic tissue surrounding the left subclavian artery (LSA) was free of malignancy (arrow). (d) Microscopically, the fibrosis surrounding the left common carotid artery (LCCA: arrow) was free from malignancy, but the tumor had infiltrated the left brachiocephalic vein (^∗^).

A 16-year-old male with neurofibromatosis type 1 was referred to our hospital for management of left superior mediastinal tumor. The surgery in this case reports is for the patient at the time of the fourth surgery for malignant peripheral nerve sheath tumor (MPNST). For the first surgery, owing to the rapid growth of the tumor and presence of dyspnea, he underwent an emergent operation through trap door thoracotomy (Figures 1(a) and 1(b)). The tumor was 123 × 120 × 88 mm in size and was originated from the left thoracic vagal nerve distal to the bifurcation of the recurrent laryngeal nerve. We could barely exfoliate the tumor from the LCCA and LSA, and it was diagnosed as MPNST with 56 mitosis cells per 20 high-power fields. Radiotherapy of total dose 60 Gy was administered to the mediastinum owing to pathologically positive margins. In the second operation, a hook approach was used to perform combined resection of the left chest wall including the 2^nd^-4^th^ ribs for locally recurrent tumor. In the third operation, a distal pancreatectomy was performed for metastasis to the pancreas. Ten months after the first operation, we observed rapid growing local recurrence in the left superior mediastinum. The recurrent tumor was not found on examination a month ago. Chest computed tomography (CT) showed that the recurrent tumor attached the left clavicular head, the first rib, upper sternal bone, the LCCA, and LSA (Figures 1(c) and 1(d)). Preoperative cranial magnetic resonance angiography showed a normal vertebral-basilar system, and a tumor measured 35 mm in size in the occipital lobe. We prioritized the operation of the thoracic tumor due to its rapid growth, and we planned an endovascular technique to avoid intraoperative fatal bleeding. We obtained written informed consent from his parents for the off-label use. An 8 mm in diameter and 60 mm in length polytetrafluoroethylene-covered stent (PTFE-CS) for biliary tract (Fluency®; Bard, Karlsruhe, Germany) was inserted in the area where the tumor was sufficiently covered from the LCCA bifurcation by cutting down at the left neck, and postdilatation was performed with a 6 mm balloon dilatation catheter. Then, other PTFE-CS, 8 mm in diameter and 60 mm in length, was inserted from LSA bifurcation. Another PTFE-CS, 8 mm in diameter and 40 mm in length, was further inserted peripherally to cover the left vertebral artery to cover the tumor, and postdilatation was performed. The procedure was complete without complications. On the next day, we performed tumor resection, combined resection of the left brachiocephalic vein and pericardium and partial resection of the left upper lobe and chest wall (from the first to third ribs, the left clavicular head, and the upper left half of the sternum). Because the tumor was firmly fixed in the left upper mediastinum due to the effect of past radiotherapy, the tumor could be barely exfoliated from the both arteries. In spite of resecting the wall of the LSA during the operation, there was no bleeding from the defect of the LSA with one-third of the circumference, and it was covered with a pericardial patch. Subsequently, the thoracic wall was reconstructed with Gore-Tex® dual mesh (Figures 2(a) and 2(b)). Pathologically, the tumor had not infiltrated into LCCA and LSA, and the surgical margin was negative (Figure 3). He was discharged on postoperative day 19 without any complication. Subsequently, cranial surgery was performed 3 months after the thoracic surgery. Six months after the operation, a local recurrence occurred in the left thoracic cavity. Pazopanib was administered, but the tumor grew. At 10 months postoperatively, tracheal stenosis due to tumor growth occurred, and a tracheal stent was placed. He was treated with trabectedin but was unsuccessful and died 11 months after the surgery.

## 3. Discussion and Conclusions

Surgical resection of a thoracic tumor near major vessels, including aorta, aortic branch vessels, and other major veins, in patients with poor general conditions is inherently challenging due to the difficulty in determining the optimal approach and the extent of surgery. Recently, stent graft-assisted thoracic surgery has expanded operative indications as a minimally invasive surgery which precludes the need for a biventricular assist device or aortic arch replacement. This is the first report of stent graft placement to the LCCA and LSA prior to the invasive tumor resection. In this case, we opted for the use of covered stent due to the following considerations. First, chest CT showed fixation of the LCCA and LSA to the tumor without obvious intravenous infiltration. This region had been irradiated with a total dose of 60 Gy. There would be a high risk of fatal intraoperative bleeding from these arteries during exfoliation of the tumor. If the tumor infiltrated these arteries, we intended to perform combined resection of the tumor with partial artery. Similar cases where partial resection of the aorta was safely performed have been reported [1, 2, 5]. Second, the patient had undergone multiple invasive surgeries over a short period of time, and his physical tolerance was decreased. Further, we had planned brain tumor resection as soon as possible after this surgery. In the present case, although we injured the LSA, the operation was successful without any bleeding complication. He safely underwent brain surgery after thoracic surgery owing to the minimal physical damage.

Covered stents for the branch vessels of the thoracic aorta have already been developed. Their authority or import depends on each country, so these could not be used in emergency. We opted for prompt surgery with use of a readily available biliary stent, because the properties of the biliary stents were considered to be similar to that of covered stents for the LCCA and LSA. We chose biliary stents for the following reasons: Being a covered stent, the specifications (blood vessel diameter/length) conforming to the target blood vessel, it has been reported to be used in endovascular treatment [6, 7], and it has already been used in endovascular treatment of other patients at our hospital. Nonetheless, we needed informed consent of the patient given the off-label use. Furthermore, risk of delayed complications such as stenosis, aneurysm formation, and thrombosis is not clear. In this case, he died 11 months after the time of fourth operation due to the progression of the primary disease, but there were no stent complications during that time. The recurrent tumor including chest wall and lung were resected immediately after stent placement, and no antiplatelet drug or anticoagulant was used. Since we planned a surgery for metastatic brain tumors, we did not use antiplatelet and anticoagulants because of the risk of bleeding. In the case which the urgency is low and a long-term prognosis is expected, it is preferable to use an indicated intravascular stent and to use antiplatelets and anticoagulants in combination considering the risk of bleeding. As the reports on long-term follow-up are few, the accumulation of future cases is necessary.

## Data Availability

The datasets supporting the conclusions of this article are included within the article.

## Abbreviations

LCCA: Left common carotid artery
LSA: Left subclavian artery
MPNST: Malignant peripheral nerve sheath tumor
CT: Computed tomography
PTFE-CS: Polytetrafluoroethylene-covered stent.
